# Moving Toward Meaningful Evaluations of Monitoring in e-Mental Health Based on the Case of a Web-Based Grief Service for Older Mourners: Mixed Methods Study

**DOI:** 10.2196/63262

**Published:** 2024-11-28

**Authors:** Lena Brandl, Stephanie Jansen-Kosterink, Jeannette Brodbeck, Sofia Jacinto, Bettina Mooser, Dirk Heylen

**Affiliations:** 1 Human Media Interaction group, University of Twente, Drienerlolaan 5, Enschede, 7522NB, Netherlands, 31 534893740; 2 Roessingh Research and Development, Enschede, Netherlands; 3 Biomedical Signals and Systems, University of Twente, Enschede, Netherlands; 4 Institute for Psychology, University of Bern, Bern, Switzerland; 5 School of Social Work, University of Applied Sciences and Arts Northwestern Switzerland, Olten, Switzerland; 6 Centro de Investigação e Intervenção Social, Instituto Universitário de Lisboa, Lisboa, Portugal

**Keywords:** e-mental health, digital mental health service, mental health, digital health, internet intervention, monitoring mental health, monitor, e-coach, coaching, grieve, mourn, old, affective states, artificial intelligence, predictive, repeatedly measured predictors in regression, fuzzy cognitive map, algorithm, AI

## Abstract

**Background:**

Artificial intelligence (AI) tools hold much promise for mental health care by increasing the scalability and accessibility of care. However, current development and evaluation practices of AI tools limit their meaningfulness for health care contexts and therefore also the practical usefulness of such tools for professionals and clients alike.

**Objective:**

The aim of this study is to demonstrate the evaluation of an AI monitoring tool that detects the need for more intensive care in a web-based grief intervention for older mourners who have lost their spouse, with the goal of moving toward meaningful evaluation of AI tools in e-mental health.

**Method:**

We leveraged the insights from three evaluation approaches: (1) the *F*_1_-score evaluated the tool’s capacity to classify user monitoring parameters as either in need of more intensive support or recommendable to continue using the web-based grief intervention as is; (2) we used linear regression to assess the predictive value of users’ monitoring parameters for clinical changes in grief, depression, and loneliness over the course of a 10-week intervention; and (3) we collected qualitative experience data from e-coaches (N=4) who incorporated the monitoring in their weekly email guidance during the 10-week intervention.

**Results:**

Based on n=174 binary recommendation decisions, the *F*_1_-score of the monitoring tool was 0.91. Due to minimal change in depression and loneliness scores after the 10-week intervention, only 1 linear regression was conducted. The difference score in grief before and after the intervention was included as a dependent variable. Participants’ (N=21) mean score on the self-report monitoring and the estimated slope of individually fitted growth curves and its standard error (ie, participants’ response pattern to the monitoring questions) were used as predictors. Only the mean monitoring score exhibited predictive value for the observed change in grief (*R*^2^=1.19, SE 0.33; *t*_16_=3.58, *P*=.002). The e-coaches appreciated the monitoring tool as an opportunity to confirm their initial impression about intervention participants, personalize their email guidance, and detect when participants’ mental health deteriorated during the intervention.

**Conclusions:**

The monitoring tool evaluated in this paper identified a need for more intensive support reasonably well in a nonclinical sample of older mourners, had some predictive value for the change in grief symptoms during a 10-week intervention, and was appreciated as an additional source of mental health information by e-coaches who supported mourners during the intervention. Each evaluation approach in this paper came with its own set of limitations, including (1) skewed class distributions in prediction tasks based on real-life health data and (2) choosing meaningful statistical analyses based on clinical trial designs that are not targeted at evaluating AI tools. However, combining multiple evaluation methods facilitates drawing meaningful conclusions about the clinical value of AI monitoring tools for their intended mental health context.

## Introduction

Artificial intelligence (AI) tools hold much promise for mental health care by increasing the scalability and accessibility of care [1]. They have the potential to identify warning signs of serious mental health problems earlier than current mental health care systems allow and deliver timely (digital) mental care, potentially preventing the full onset of mental health disorders or limiting the severity with which they impair people’s lives [1,2]. For example, Sakal et al [3] described the development and evaluation of an AI-based screening tool for geriatric depression in Chinese older adults. Taking into account cultural response biases to traditional depression screening tools, the tool focused on less emotionally sensitive demographic and quality of life predictors such as health status compared to 3 years ago, hearing status, income, and average hours of sleep per night in the previous month. The tool was found to perform well during validation and the authors explained the importance of the nonsensitive nature of the questions used by the screening tool for early detection of geriatric depression in the Chinese aging population. The tool represents a means for Chinese public health officials to fight the growing mental health treatment gap in the country. Likewise, Zhang et al [4] leveraged AI to extensively analyze behavior-related and physiological risk factors for suicide in middle- and older-aged individuals who participated in the UK Biobank population-based cohort that was recruited between 2006 and 2010. The use of AI and advanced statistical tools enabled the authors to systematically identify and rank 246 behavior-related and 200 physiological factors and identify 58 robust predictors for suicide risk. The authors explained that the gained insights unravel new potential avenues for targeted suicide prevention.

Despite such promising examples of how AI tools can contribute to increasing the scalability, accessibility, and effectiveness of mental health care, AI tools are currently still considered to be in a proof-of-concept stage rather than currently having a clinical impact on mental health care [5]. Tornero-Costa et al [6] described a mismatch between clinical trial designs that are common in mental health care and desired data qualities for AI development, which are often difficult to reconcile in terms of time, money, and human resources. AI tools and clinical trials have fundamentally diverged data sampling considerations, specifically concerning exclusion criteria that are common in clinical trials to limit the influence of confounders on tested clinical outcomes, or due to safety considerations. However, given large enough sample sizes, confounders improve the generalizability of AI models, which makes them a necessary element in any representative dataset. In mental health care, AI tools are currently often developed in retrospection as secondary outcomes of clinical trials and are based on clinical data collected for purposes other than model development [6].

In addition, current AI model engineering approaches for mental health are criticized for their focus on perfecting model performance without providing practical clinical value [7,8]. Whiting and Fazel [7] explained in their recent clinical meta review on the accuracy of prediction models for detecting suicide risk that only a few models are developed with independent clinical validation or piloting in mind. Model developers tend to neglect the clinical meaning of the association between predictors and model outcomes and are not transparent regarding the decision-making process leading to the selection of model parameters [6]. Furthermore, current practices favor model evaluation metrics such as predictive accuracy without explaining how they are linked to a clinical decision. In the specific context of suicide risk detection, the authors advocate that prediction models should be compared to unstructured clinical assessments of suicide risk to investigate the incremental benefit of these tools in supporting clinician decision-making. Ultimately, suicide prediction is challenging for both data-driven prediction models and clinical practitioners, as is any mental health prediction task. To build AI tools in mental health care with a clinical impact, we need to start developing and evaluating models whose outcomes can be clearly linked to clinical decision-making and their roles in clinical practice should be well-defined.

In this paper, we evaluate a mental health monitoring tool in an e-mental health service for older mourners by combining the insights from 3 evaluation approaches. We encountered some challenges that are common in AI evaluation studies and showcase how these affect the clinical meaningfulness of our obtained results. We exemplify the need for AI tools in mental health care to go beyond classical AI evaluation metrics and statistical approaches in clinical research to have an impact. The next section briefly introduces the monitoring tool and the e-mental health service in which it is embedded before describing our evaluation approach in more detail. The e-mental health service for which the monitoring tool was developed supports older mourners in processing the loss of their spouse. We conclude with a discussion of the encountered evaluation challenges and some suggestions on how to move the development of impactful AI tools in mental health care forward.

## Methods

### Background: The Monitoring Tool

The monitoring tool that we evaluated in this study is implemented in a web-based grief service for older mourners who have lost their spouse. The grief service consists of 10 content modules (eg, unraveling myths and truths about grief) and exercises and activity suggestions that help the mourner process the loss and foster positive mental and physical well-being (eg, writing a farewell letter to the deceased spouse, reconnecting with one’s hobbies) [9]. The monitoring tool complements the service with a biweekly mental health self-check and by analyzing whether it is advisable for the user to seek offline (professional) support. It has 2 components: a mental health user profile and a decision-making component. The mental health user profile consists of 2 self-report questionnaires, an initial risk assessment (IRA) and a continuous risk assessment (CRA). The IRA represents an initial assessment of the user’s affective state and grief symptoms when they start using the web-based grief service and controls for risk factors such as whether the loss has been violent (eg, their partner committed suicide). The CRA assesses the extent to which the mourner experiences psychological suffering. The decision-making component consists of a set of rules that determines whether the user exceeds a suicidal threshold; it also includes a fuzzy cognitive map (FCM) decision algorithm. It arrives at the decision to display either a recommendation to seek offline support or an encouragement to continue using the grief service as is. Filling in the CRA is optional; the grief program can be used without it. The development of the monitoring tool—including its parameter selection, the construction of the 2 monitoring questionnaires (IRA and CRA), and an initial error analysis based on fictitious scenarios—is described in detail in Brandl et al [10].

### Evaluation Context: Randomized Controlled Trial and e-Coach Focus Group

The current evaluation of the mental health monitoring tool is based on an ongoing randomized controlled trial (RCT) that started in March 2022 in Switzerland [9]. The primary aim of the RCT is to investigate the clinical efficacy of the previously described web-based grief service, while the secondary aim is to examine which delivery format of the web-based grief service (standardized vs self-tailored) is associated with better clinical outcomes. At the time of writing this paper, the RCT is ongoing until the needed sample size to test the 2 delivery formats of the service is achieved. Our evaluation approach uses the data of older mourners (≥60 years old) who participated in the RCT. Participants were recruited from the general population and had experienced the loss of their partner at least 1 month before the RCT. A more extensive list of inclusion and exclusion criteria can be found in the dedicated study protocol of the RCT [9]. During the RCT, 4 e-coaches provided guidance in the form of a weekly email with short, personalized feedback and support. The e-coaches were encouraged to include participants’ self-reported mood and therapeutic progress and the outcome of the monitoring tool in the guidance that they provided.

### Evaluation Approach

#### Overview

To evaluate the monitoring tool, we did the following: (1) assessed the classification performance of the monitoring decision algorithm using the *F*_1_-metric; (2) investigated the predictive value of participants’ monitoring responses for their clinical change in grief, depression, and loneliness after the 10-week RCT; and (3) collected qualitative user experience data to explore the tool’s suitability for clinical practice from trained e-coaches who used the monitoring for their work during the RCT. For the first step, the classification performance was assessed using ground truth classification labels provided by the e-coaches for the tool’s binary outcome (recommendation to seek support vs encouragement to continue using the grief service as is). The e-coaches determined the ground truth labels based on their professional assessment given the participant’s progress in the e-mental health service, their biweekly monitoring responses, weekly email exchanges with the participant, and a clinical interview at the beginning of the RCT. The monitoring’s suggested classification was visible to the e-coaches alongside participants’ raw monitoring responses to facilitate the e-coach’s understanding of the classification. If the e-coach’s assessment diverged from the outcome of the monitoring tool, they provided a brief textual explanation about their rationale. The ground truth labels for the monitoring predictions were provided by the e-coaches upon request at the time of conducting this analysis. For the second step, we explored the predictive value of the CRA by relating CRA scores to the difference in clinical measurements before and after the RCT. For the third step, we focused on the e-coaches’ experiences with the monitoring tool during the RCT. A web-based focus group was conducted in which the 4 e-coaches discussed how they used the monitoring tool in their role as e-coaches, how they experienced having the tool at their disposal, and how they think such a tool could be most useful for mourners who use the web-based grief service and for e-coaches such as themselves.

#### Measures

The CRA is a multidimensional scale that measures hopelessness, grief symptoms, social isolation, and psychological crisis with 2 items each on a 4-point Likert scale. The items assess the frequency of emotional suffering in the past 2 weeks ranging from 0 (Not at all) to 3 (Every day). The CRA also measures therapeutic progress on a 4-point Likert scale ranging from 0 (Strongly disagree) to 3 (Strongly agree). The CRA serves as input for the FCM algorithm as part of the decision-making process in the monitoring model. Its development is described in more detail in Brandl et al [10]. A copy of the scale is included in Multimedia Appendix 1. For this study, the CRA scores of RCT participants were retrieved from data logs of the grief program. The three clinical measures (grief, loneliness, depression) were assessed at three measurement moments during the RCT via a web-based surveying tool: (1) prior to starting the web-based grief program (*t*_0_), (2) after completing the 10-week intervention (*t*_1_), and (3) twenty weeks after starting the intervention program (*t*_2_). For the current evaluation of the monitoring tool, we only take the first 2 measurement moments into account. The clinical measures include an assessment of the mourner’s (1) grief symptoms using the Texas Revised Inventory of Grief [11], (2) depressive symptoms using the Patient Health Questionnaire-9 [12], and (3) loneliness via the de Jong Gierveld Loneliness Scale [13,14].

#### Data Inclusion

For the evaluation of the classification performance, our first approach, we included any monitoring decision for which the e-coaches provided a ground truth label. For assessing the predictive value of the CRA for clinical change during the RCT, however, we only included participants who had (1) completed the 10-week intervention and (2) filled in the clinical measurements (depression, grief, loneliness) at baseline and 10 weeks after starting the intervention. We did not expect the delivery format of the grief program (self-tailored vs standardized) or the fact that participants in the waitlist control condition received access to the intervention only after 12 weeks to impact the decisions of the monitoring algorithm. Likewise, we did not expect the delivery format or the waitlist control condition to affect the relation between how participants filled in the CRA and the clinical outcomes. Therefore, we included participants from all arms of the RCT in this analysis. Specifically, we included CRA scores from RCT weeks 2-10. In week 2, the CRA was administered for the first time.

### Ethical Considerations

The RCT based on which we evaluated the current AI monitoring tool of a web-based grief service received medical ethical approval from the Medical Ethical Committee of Northwestern and Central Switzerland (Business Administration System for Ethics Committees number 2021‐02221) and is registered at ClinicalTrials.gov (NCT0528004). All older mourners who participated in the RCT signed an informed consent form that was approved by the Medical Ethical Committee of Northwestern and Central Switzerland, allowing the secondary analysis of their monitoring data for the purpose of evaluating the web-based grief service with no further consent required. In addition, the e-coaches provided written informed consent prior to participation in the focus group. All analyses involving the data of RCT subjects were conducted on an anonymized dataset where each participant was represented by an arbitrary code that is not related to their identity. The e-coach focus group was recorded and automatically transcribed using Microsoft Teams. After checking the correctness of the automatic transcription, the recording was deleted, and the transcription was deidentified. All subsequent analyses were conducted using the deidentified focus group transcription. Participants did not receive (financial) compensation for participating in this research. We refer the reader to the dedicated RCT study protocol for more detailed information about its ethical review process [9].

### Data Analyses

#### Analysis I: Classification Evaluation

Our data were sampled from a nonclinical population. Therefore, we expected few (true) help-seeking recommendations in the sample. This has implications for choosing an appropriate classification evaluation metric [15,16]. Regarding terminology, in the binary classification problem at hand (recommending to seek offline support versus recommending to continue using the service as is), we chose the less frequent class, recommending help-seeking, as the positive outcome class and recommending to continue using the service as the negative class, as recommended for imbalanced classification problems [16]. The *F*-measure is used when there is no clear preference for either minimizing false positives (someone receives an unjustified recommendation to seek support) or false negatives (someone who needs support does not receive a recommendation to seek support) because both are regarded as equally important for determining the classifier’s performance. The *F*_1_-score is the harmonic mean between the true positive rate (recall) of a classifier and its precision:


(1) recall=truepositives/(truepositives+falsenegatives)

(2) precision=truepositives/(truepositives+falsepositives)

(3) $$F_{1}-score=2*(recall*precision)\;/\;(recall+precision)$$

The *F*_1_-score is bounded to the interval [0*,*1], where 1 represents maximum precision and recall and 0 represents zero precision and recall. All calculations were performed using Python 3.11 [17].

#### Analysis II: Predicting Clinical Change Using Monitoring Measurements

The reliability of the CRA questionnaire was assessed using Spearman-Brown *ρ_SP_* coefficients for its five 2-item subscales: psychological crisis, hopelessness, grief symptoms, social isolation, and therapeutic progress [18]. As pointed out by Tavakol and Dennick [19], if a scale measures several constructs, it is recommended that reliability is assessed separately for each construct. Since each CRA construct is measured using 2 items, Spearman-Brown coefficients were deemed the most appropriate method for assessing reliability [18]. To investigate the relation between the CRA and the clinical outcomes of the RCT, we conducted 3 linear regression analyses, with the difference in clinical outcomes before and after the grief intervention as the dependent variable and the parameters of an individually fitted linear growth curve (the estimate of the linear coefficient and its standard error, ie, the slope of the linear curve) and mean CRA scores as independent variables, as suggested by Welten et al [20] for repeatedly measured predictors. Analyses were performed using Python 3.11 and R (version 4.3.2; R Foundation for Statistical Computing) [21].

#### Analysis III: e-Coaches’ Experience With the Monitoring Tool

An inductive coding scheme was developed and applied in ATLAS.ti [22] to the transcript of the e-coaches’ focus group about their experiences with the monitoring tool during the RCT. The coding scheme was developed and applied by one researcher and verified by a second researcher. Any discrepancies were discussed until agreement was reached. An exemplary code is *monitoringExp*, which summarizes experiences and thoughts that the e-coaches had about having the monitoring at their disposal.

## Results

### Analysis I: Classification Evaluation

#### Participants

The data of 44 RCT participants were included in the assessment of the classification performance of the monitoring module. On average, these 44 participants filled in 4.02 (SD 2.2) of the 5 biweekly CRA questionnaires during the 10-week intervention, amounting to 174 monitoring decisions that were labeled by hand by the e-coaches.

#### Confusion Matrix and *F*_1_-score

Table 1 shows the confusion matrix for the monitoring algorithm’s decision-making. Most labeled monitoring decisions (n=168) were true negatives, reflecting that detecting the need for professional intervention in a web-based grief service is an extremely imbalanced classification problem. Taking a closer look at the only false negative classification, the e-coach explains that they disagreed with not recommending additional support because the participant indicated an exacerbation of psychosomatic symptoms (eg, heart pounding) in their email exchange with the e-coach as well as a lack of future perspective. The FCM does not include psychosomatic symptoms in its decision-making, but it does include a measure of “lack of future perspective” (hopelessness). Four of the 5 true positives occurred in the initial monitoring assessment where additional risk factors are assessed, such as a recent inpatient treatment for a psychological condition. Only 2 of the 3 participants exhibited such risk factors; those were also named by the e-coach as reasons why they agreed with the recommendation to seek additional support. The remaining true positives represented moments of elevated emotional suffering as reflected by the participants’ monitoring responses in the CRA. The monitoring algorithm’s *F*_1_-score was 0.91.

**Table 1.** Confusion matrix of the monitoring decision algorithm that either recommends help-seeking or to continue using the mental health service with no change.

	Positive class (help-seeking recommendation)	Negative class (no help-seeking recommendation)
True prediction	5	168
False prediction	0	1

### Analysis II: Predictive Value CRA for Clinical Change

#### Participants

Since we only included participants who had both completed the 10-week intervention and filled in the clinical measurements (depression, grief, loneliness) at baseline and 10 weeks after starting the intervention, 21 participants were included in the analysis that assesses the predictive value of the CRA. Participants’ mean age was 60.1 (SD 11.4) years. Of the 21 participants, 18 were female and 3 were male. Using Spearman-Brown coefficients *ρ_SP_* to assess the reliability of the 5 CRA subscales resulted in *ρ_SP_*=0.74 for the hopelessness subscale, *ρ_SP_*=0.70 for the grief symptoms and therapeutic progress subscales, *ρ_SP_*=0.66 for the psychological crisis construct, and *ρ_SP_*=0.14 for the social isolation subscale. *ρ_SP_* scores between 0.5 and 0.7 are considered fair and scores between 0.7 and 0.9 are considered good [23]. Further investigation into the low Spearman-Brown coefficient *ρ_SP_* for the social isolation construct revealed that the 2 items in the subscale correlated poorly (Pearson *r*=0.08).

#### Linear Regression Analysis

**Figure 1.**
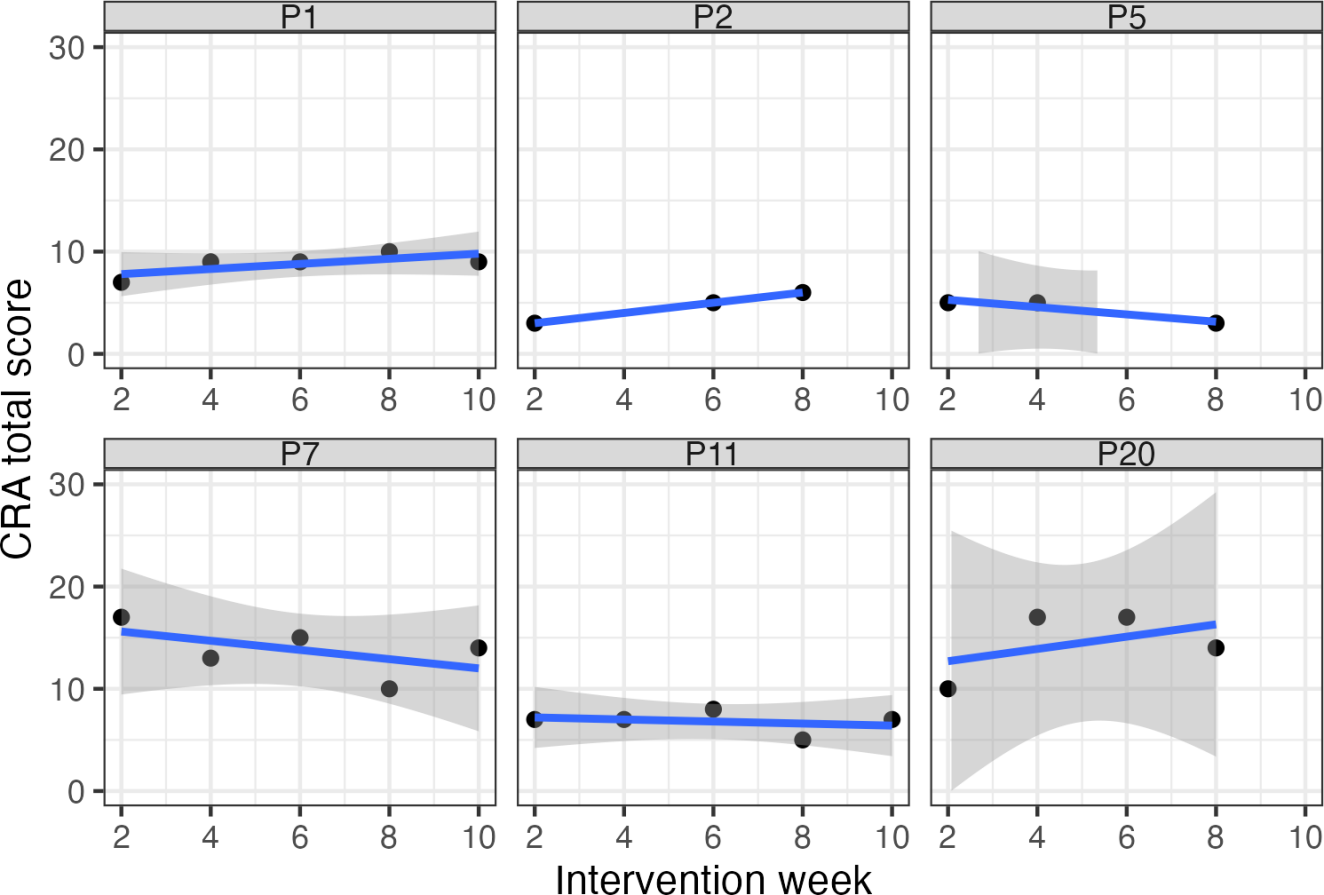
Subset of fitted linear individual growth curves that served as predictor variables in the regression analysis. CRA: continuous risk assessment.

Table 2 shows the descriptives of the dependent and independent variables in the regression analysis. Depression and loneliness measurements before the RCT (*t*_0_) and after the RCT (*t*_1_) differed little, making it difficult to reliably fit a model using either as the dependent variable. We therefore decided to only conduct 1 regression analysis, with the difference in grief scores before and after the RCT as the dependent variable. Not everyone filled in the CRA regularly, resulting in n=84 CRA measurements that were included in the analysis. Figure 1 shows a subset of the fitted individual growth curves; the entire set is included in Multimedia Appendix 2. Table 3 summarizes the results of the linear regression analysis with the difference in grief before and after the RCT as the dependent variable and individual CRA growth curves and CRA means as predictors. Overall, the regression model fit the observed data well (*R*^2^=0.45; *F*_3,16_=4.42, *P*=.019). Neither the slope of the individually fitted CRA curves (*R*^2^=−1.18, SE 2.21; *t*_16_=−0.54, *P*=.60) nor their standard error (*R*^2^=−4.45, SE 3.03; *t*_16_=−1.47, *P*=.16) predicted how grief symptoms changed during the RCT. CRA mean scores did have predictive value for how mourners’ grief scores changed during the intervention (*R*^2^=1.19, SE 0.33; *t*_16_=3.58, *P*=.002). We checked statistical assumptions visually, including normality and homoscedasticity of residuals, and found none to be violated.

**Table 2.** Descriptives of the independent and dependent variables in the regression analysis.

Variable		Mean (SD)	Median (min, max)	Scale range [reference]
Continuous risk assessment total score (n=84)^a^		8.68 (4.16)	9.0 (1.0, 21.0)	0‐24 [10]
***t***_**1**_ **–** ***t***_**0**_ **difference (N=21)**				
	Grief	−2.9 (5.37)	−2.0 (−12.0, 6.0)	5-80 [9,24]
	Depression	−0.76 (2.83)	0.0 (−7.0, 5.0)	0-27 [12]
	Loneliness	−0.29 (1.27)	0.0 (−3.0, 3.0)	0-6 [25]

a Of 105 possible continuous risk assessment measurements, 21 (20%) were missing.

**Table 3.** Summary of the linear regression analysis with individually fitted growth curve parameters and continuous risk assessment mean scores as independent and difference in grief before and after the 10-week web-based grief intervention as dependent variables.

	*R*^2^ (95% CI)	*t* test (*df*)	*P* value
Intercept	−11.37 (−16.82 to −5.92)	−4.42 (16)	<.001
Slope of growth curve	−1.18 (−5.87 to 3.5)	−0.54 (16)	.60
Standard error of the growth curve slope	−4.45 (−10.88 to 1.98)	−1.47 (16)	.16
Continuous risk assessment mean	1.19 (0.48 to 1.89)	3.58 (16)	.002

### Analysis III: e-Coaches’ Experience With the Monitoring Tool

All e-coaches that provided guidance during the RCT (N=4) participated in the online focus group to discuss their experience with the monitoring tool. The e-coach team consisted of 1 trained psychological therapist and 3 final-year clinical psychology students who partook in the e-coaching as part of their training. Their mean age was 26.9 (SD 2.69) years. All e-coaches were female. The final-year students were trained to provide email guidance and were closely supervised by a trained psychotherapist. Before the start of the RCT, the e-coaches discussed how they would use the monitoring tool to check on participants’ health regularly. To determine a deterioration in a participant’s mental health, the e-coaches took into account the mourner’s CRA responses, recommendations suggested by the monitoring algorithm, their impression of the mourner from the clinical interview (eg, knowledge about the death anniversary date of the deceased), and the mourner’s weekly email communication, if available. The e-coaches weighted recent CRA responses most and whether there was a pattern in the response behavior. One e-coach explained that they incorporated the monitoring responses into their weekly guidance emails for unresponsive participants to personalize their contact with them. All e-coaches confirmed that they used the monitoring to confirm their existing impressions of participants:

I think we regarded it as a kind of a safety option, to check how people are feeling and how it aligns with our impression of the person and the remaining contact we have with them. And for us to reflect, did we overlook anything or forget to ask anything?

[e-coach 1]

The e-coaches unanimously experienced having another source of information as helpful, especially for participants who otherwise communicated little with them during the RCT. The e-coaches experienced being able to monitor participants’ progress with the grief service as supportive and reassuring:

Whether they [the mourner] made progress or deteriorated, a kind of support for recognizing if anything were to happen. Maybe if they [the mourner] did not tell us, to have another chance at detecting it.

[e-coach 3]

Another e-coach agreed that they were curious about how participants responded in the monitoring, especially when mourners worked on intervention content that the e-coaches knew to be challenging for some mourners, such as writing a farewell letter to the deceased spouse. For future versions of the monitoring tool, the e-coaches suggested providing feedback directly to the mourner, such as regular written summaries and recommendations to seek additional support in times of crisis. This could support the mourner’s reflection about their affective states and encourage them to seek offline support proactively instead of waiting until an e-coach advises them to seek support. In addition, the e-coaches expressed that they would prefer to receive warning messages (eg, via SMS text message) whenever the condition of a mourner deteriorated drastically to facilitate immediate intervention.

## Discussion

### Principal Findings

This study is situated in the rapidly emerging field of AI tools for mental health care and evaluated a monitoring module in a web-based grief intervention for older mourners with the aim of guiding them to offline support if their mental health deteriorated. We leveraged the insights from 3 evaluation approaches and encountered 3 main challenges when trying to come up with satisfactory and clear conclusions about “how well” a monitoring module such as the one evaluated in this study performs.

First, many clinical classification problems are (extremely) imbalanced, meaning that the class for which correct classification is crucial (eg, recommending help-seeking, detecting a tumor) is underrepresented in real-life datasets [15]. Although there are evaluation metrics, including the *F*_1_-metric [16,26] that we used in this study, that can mitigate class imbalance to some extent [27], the clinical meaningfulness of obtained results should still be appraised critically. Complementing the evaluation with qualitative accounts from clinical practice is in line with Whiting and Fazel’s [7] suggestion to consider the incremental benefit of AI tools in clinical practice and stress that any thorough evaluation of a monitoring tool should go beyond quantifiable accuracies and statistics. A monitoring tool that does not match the needs and preferences of its users and the clinical context in which it is used will ultimately not be used, regardless of its classification performance [28]. The qualitative evaluation of the tool revealed that the e-coaches envisioned the tool not only as a regular mental health check but also as an emergency detection tool for short-term psychological crisis. A more appropriate approach to evaluating the latter would be to investigate CRA measures around an episode of psychological crisis. However, the low prevalence of psychological crisis in our data makes any evaluation targeted at detecting emergencies impossible. The tool should be evaluated in a clinical sample in which short-term psychological crisis is expected to arise more frequently to investigate its suitability as an emergency detection tool.

Finding appropriate statistical approaches to evaluate AI tools for clinical practice using real-life mental health data represents a second challenge. Despite the mixed results obtained in this study, we argue that statistical approaches that allow for the explicit modeling of individual differences should receive more attention in future evaluations of AI tools in mental health care. Individual growth curve predictors are recommended when distinct developmental patterns are expected across outcome groups—in our case, we expected distinct patterns for each individual participant [20]. Grief and its experienced intensity are inherently individual [29], suggesting from a clinical point of view that individually optimized growth curves are a suitable means of analysis. In this study, individually fitted CRA growth curves captured participants’ response patterns variably well. Participants’ response patterns may require more complex functions (eg, quadratic, cubic) than linearly fitted curves. Another reason why the estimated growth curves fit participants’ response patterns variably well is missing values [20]. The reliability of the underlying measurement tool likewise impacts the fit of estimated growth curves. The social isolation subscale needs revision, as its 2 items were poorly correlated. The 2 items capture 2 different dimensions of being socially isolated: the feeling of being a burden to others and active social withdrawal behavior. It is difficult to reliably assess 2 dimensions of a construct using only 2 items. The construction of 2-item scales is generally discouraged in terms of reliability [18]; however, to limit the burden of filling in mental health checks regularly as part of a digital mental health service, short self-assessment tools are needed. In this context, incorporating less obtrusive assessment methods in digital mental health services, including sensing technologies [30] and natural language processing [31], to complement self-report monitoring of clients’ mental states should be considered in the future.

To move toward well-developed monitoring systems in e-mental health, we recommend clear and early decision-making about (1) the responsibilities of the monitoring tool in the e-mental health application (and which responsibilities the tool does not have) and (2) what it takes to evaluate the tool in a satisfactory way so that it can live up to these responsibilities and contribute in a meaningful way to clinical practice. Currently, AI tools are often developed as secondary goals to the development of a new e-mental health application [6], which represents the third identified challenge since it limits time and effort invested into their development and evaluation. Extracting clinically meaningful results using common methods for evaluating AI tools is complex. Hence, such tools cannot afford ambiguities regarding their capabilities and responsibilities that further complicate the evaluation process.

### Limitations

This study has limitations. First, the e-coaches that provided the ground truth labels for assessing the classification performance of the monitoring tool had access to the tool’s suggested decisions at the time of labeling participants’ monitoring response patterns as either “advisable to seek support” or “fine to continue using the grief service as is.” Having access to the tool’s suggestions may have biased the e-coaches’ ground truth labels in favor of the monitoring tool. However, the ground truth labels were provided by the coaches upon request at the time of conducting the analyses in this study, after RCT participants that were included in this study had completed the 10-week grief intervention. Therefore, in practice, the e-coaches revisited participants’ monitoring responses retrospectively, as well as their own initial decision-making during participants’ participation in the RCT. The retrospective nature of the labeling task likely limited the potentially introduced bias because the e-coaches had the knowledge of the participants’ trial outcome at their disposal, solidifying the truth of the provided labels. With regard to providing clinically meaningful insights, a second limitation of this study is the small sample size in the regression analysis and the small number of psychological crisis events in the classification evaluation analysis that we mentioned earlier. Any (clinical) conclusions based on the obtained results should be drawn with caution.

### Conclusion

In recent years, the demand for high-quality and accessible mental health care has been increasing. Digital mental health self-help services have the potential to support today’s health care systems in meeting care demands and their potential is further increased by leveraging the benefits of emerging AI tools, including monitoring tools that track users’ affective states and guide them toward offline support if their mental health warrants professional intervention. Such AI tools come with challenges that must be addressed systematically before they have an impact in clinical practice. These challenges include finding meaningful evaluation approaches in the face of (1) (extremely) imbalanced real-life clinical datasets, (2) ambiguous demands and expectations regarding the capabilities and responsibilities of such tools in e-mental health, and (3) priority misalignments between evaluation approaches for AI tools and the overarching goals of clinical trials in which their evaluation is usually embedded. We hope to contribute to an enhanced awareness about these challenges and to the development of evaluation approaches for AI tools in e-mental health that facilitate their introduction into clinical practice.

## Supplementary material

10.2196/63262 Multimedia Appendix 1 Continuous risk assessment questionnaire.

10.2196/63262 Multimedia Appendix 2 Regression analysis: individual continuous risk assessment growth curves.

## Abbreviations

AI: artificial intelligence
CRA: continuous risk assessment
FCM: fuzzy cognitive map
IRA: initial risk assessment
RCT: randomized controlled trial
