# Supplementary material for: Moving Toward Meaningful Evaluations of Monitoring in e-Mental Health Based on the Case of a Web-Based Grief Service for Older Mourners: Mixed Methods Study
Source: JMIR Form Res. 2024 Nov 28;8:e63262. doi: 10.2196/63262 (PMC11620699; doi:10.2196/63262)
Supplement: Multimedia Appendix 1 [file formative-v8-e63262-s001.docx]

## Appendix 1. Continuous Risk Assessment (CRA) questionnaire

Crisis detection

1. In the past 2 weeks, how often have you felt impaired in your daily life or experienced severe emotional distress which made it difficult to think of other things?

□ Not at all

□ A few days

□ More than half the days

□ (Nearly) every day

1. In the past 2 weeks, how often have you felt that you need extra support to cope with everyday life or getting through the day?

□ Not at all

□ A few days

□ More than half the days

□ (Nearly) every day

Depressive symptom: Hopelessness

1. In the past 2 weeks, how often have you given up because your future feels dark, and seems to only get darker?

□ Not at all

□ A few days

□ More than half the days

□ (Nearly) every day

1. In the past 2 weeks, how often have you felt that all that awaits you in your future is emptiness, loneliness or suffering and that there is nothing you can do about it?

□ Not at all

□ A few days

□ More than half the days

□ (Nearly) every day

Grief symptoms

1. In the past 2 weeks, how often have you felt impaired in your daily activities by intense feelings of emotional pain or suffering related to your grief?

□ Not at all

□ A few days

□ More than half the days

□ (Nearly) every day

1. In the past 2 weeks, how often have you felt impaired in your daily activities by feeling stunned or dazed by your loss?

□ Not at all

□ A few days

□ More than half the days

□ (Nearly) every day

Suicidality

- - 1. In the past 2 weeks, I have contemplated to commit suicide.

□ Yes

□ No

**If yes**, adapted Scale for Suicide Ideation (SSI) items 12 through 16 assess plans, preparations, and resolve to make a suicide attempt:

- - 1. In the past 2 weeks, I have considered a plan for a suicide attempt.

□ No, I have not worked out a plan.

□ Yes, but the details of the plan are not worked out.

□ Yes, and the details of the plan are well worked out.

- - 1. In the past 2 weeks, I felt that there was an opening for a suicide attempt or a suicide plan that I thought about was available.

| □ | The contemplated plan is not available; there is no opportunity. |
| --- | --- |
| □ | The contemplated plan would take time and/or effort and it is not readily available. I don’t see an opportunity. |
| □ | The contemplated plan is available or there is another opportunity to commit suicide. |
| □ | I anticipate that in the future, the contemplated plan will be available or there will be an opportunity. |

- - 1. In the past 2 weeks, I have considered how capable I am to commit suicide.

□ I am certain that I have **no** courage and/or **no** competence to commit suicide.

□ I am uncertain of my courage and/or competence to commit suicide.

□ I am certain that I have courage and/or competence to commit suicide.

- - 1. In the past 2 weeks, I have engaged in actual preparation for the contemplated suicide plan.

□ No, I did no preparation at all.

□ I started to prepare the contemplated suicide plan.

□ I have completed preparing the contemplated suicide plan.

Social isolation

- 1. In the past 2 weeks, how often have you been thinking that the people in your life would be better off if you were gone?

□ Not at all

□ A few days

□ More than half the days

□ (Nearly) every day

- 1. In the past 2 weeks, I have avoided getting in touch with friends and family.

□ Not at all

□ A few days

□ More than half the days

□ (Nearly) every day

Therapeutic progress

- - 1. In the past 2 weeks, I have had positive experiences in addressing my problems and/or I have gained important insights.

□ Very strongly disagree

□ Disagree

□ Agree

□ Very strongly agree

- - 1. In the past 2 weeks, I generally have been feeling better.

□ Very strongly disagree

□ Disagree

□ Agree

□ Very strongly agree
