# Supplementary material for: Moving Toward Meaningful Evaluations of Monitoring in e-Mental Health Based on the Case of a Web-Based Grief Service for Older Mourners: Mixed Methods Study
Source: JMIR Form Res. 2024 Nov 28;8:e63262. doi: 10.2196/63262 (PMC11620699; doi:10.2196/63262)
Supplement: Multimedia Appendix 2 [file formative-v8-e63262-s002.docx]

## Appendix 2. Regression analysis: individual CRA growth curves


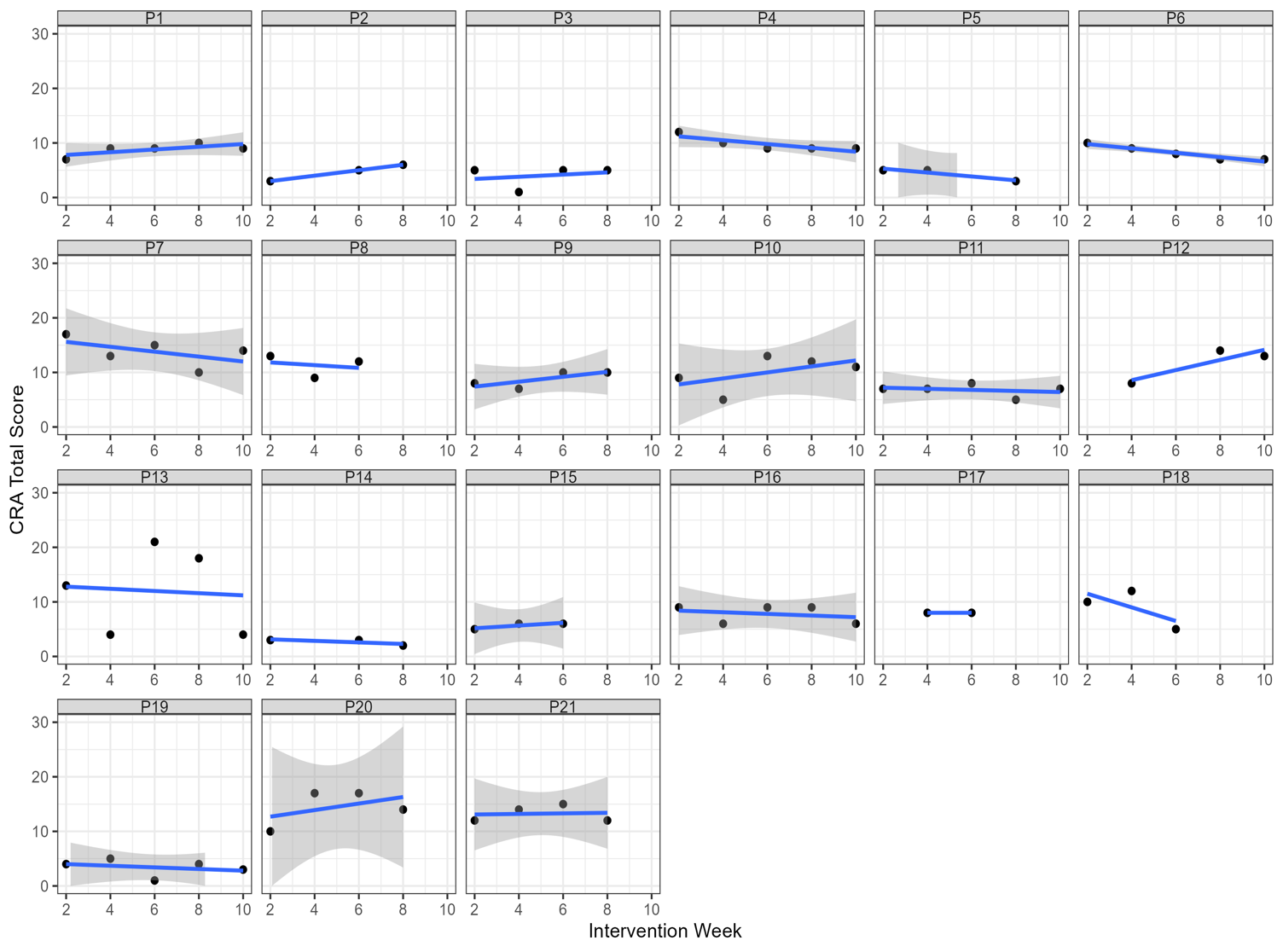


Figure 2. Fitted linear individual growth curves with the intervention week as predictor and continuous risk assessment (CRA) total scores as dependent variable. Growth curves serve as predictor variables in the regression analysis.
